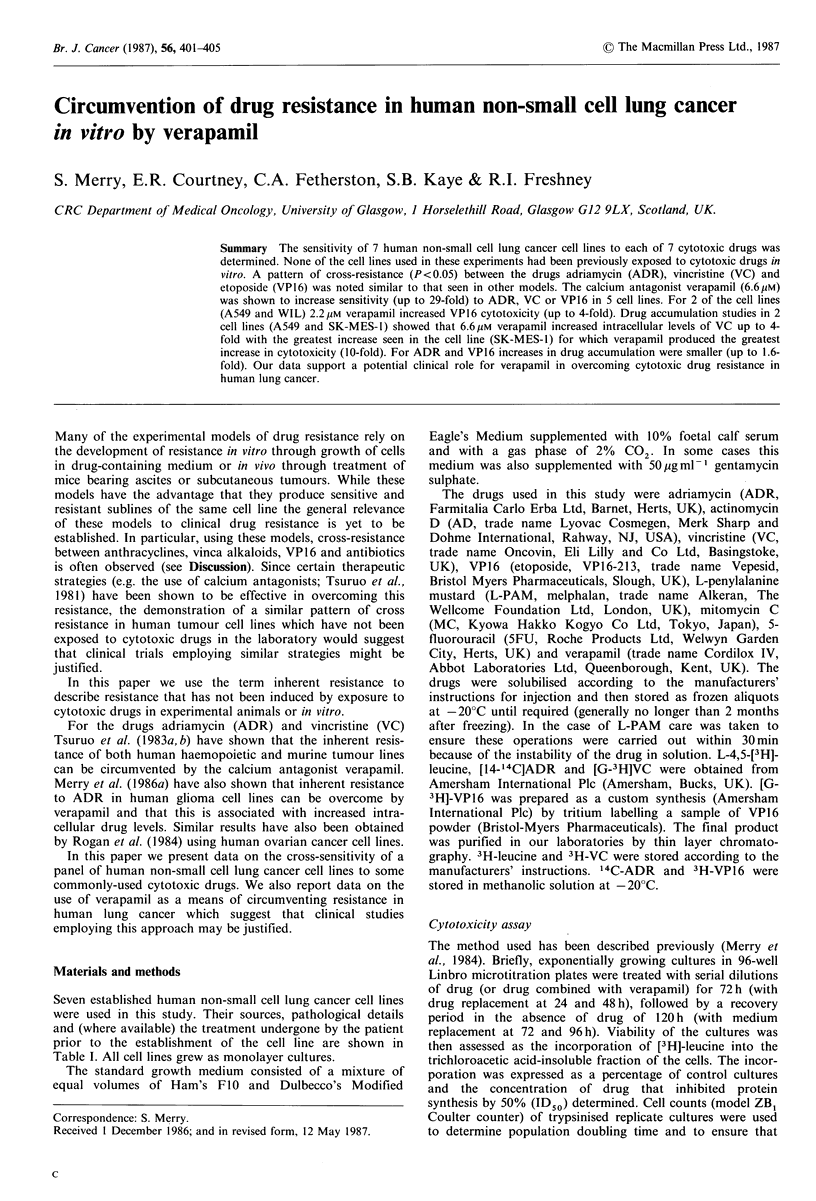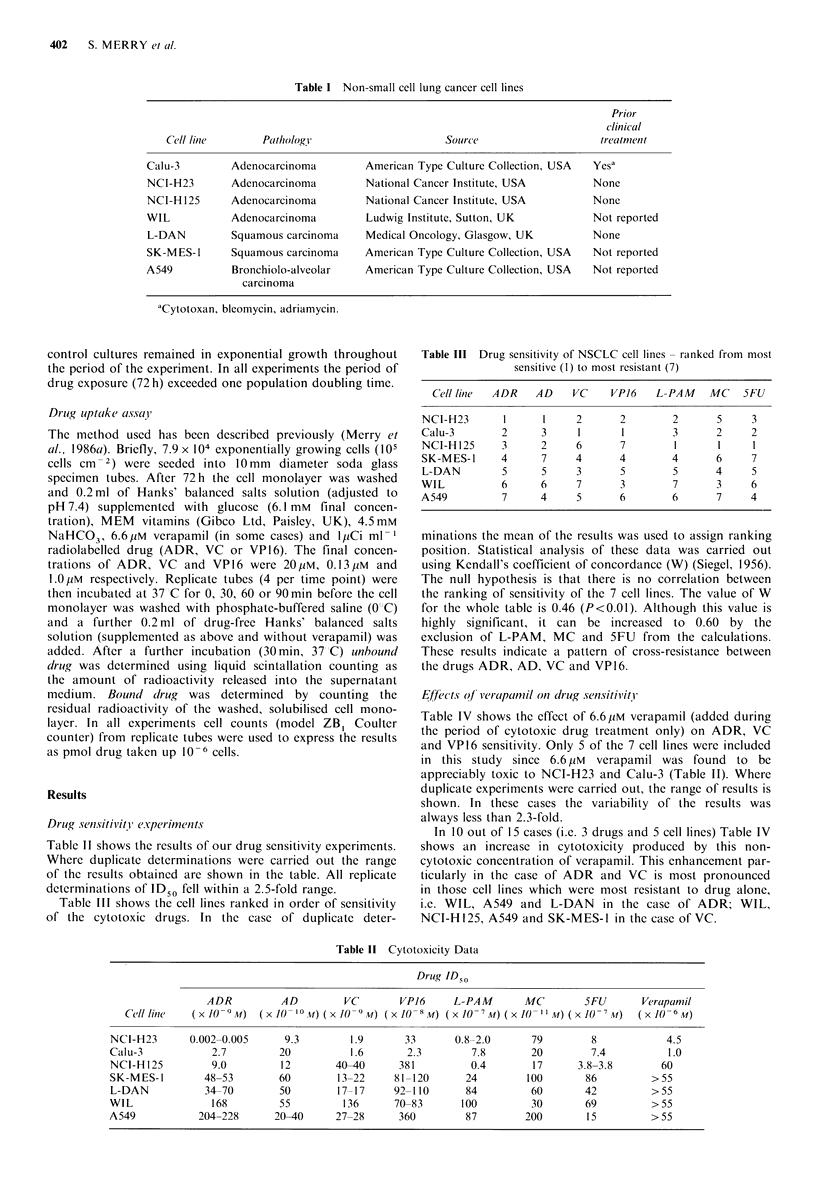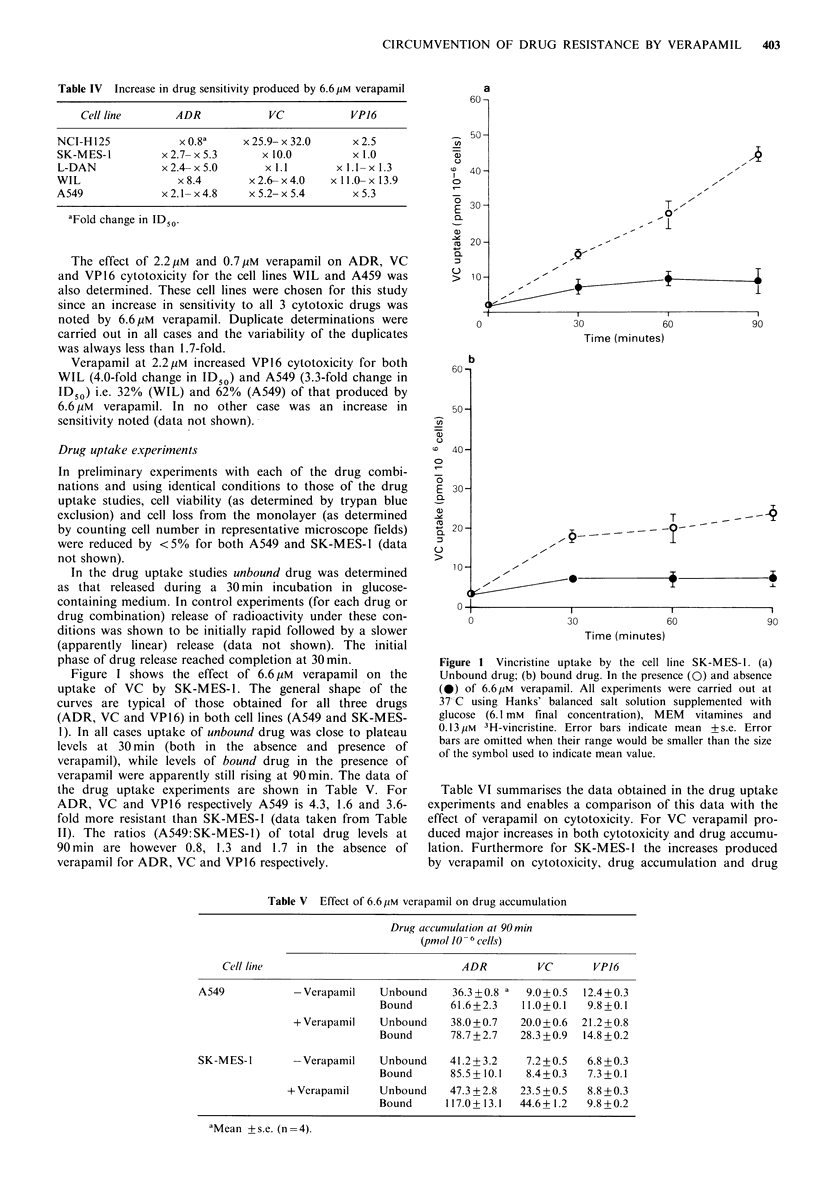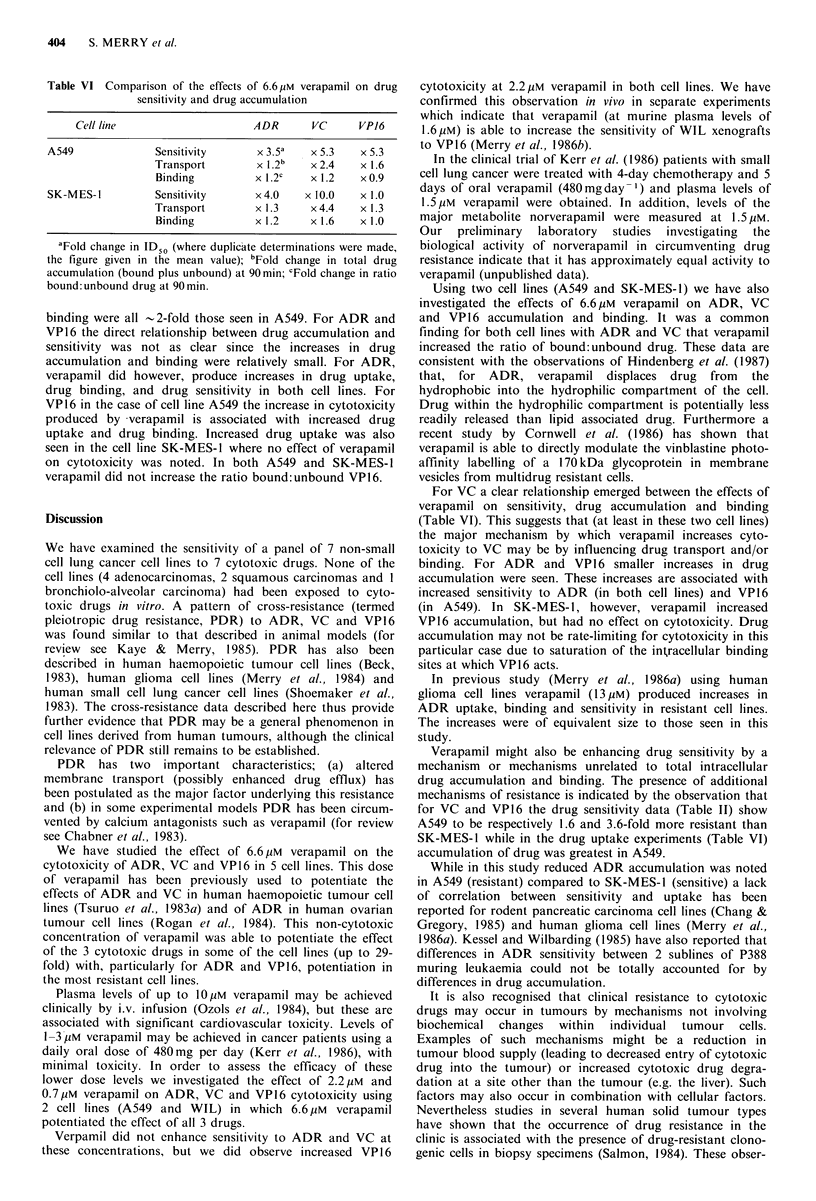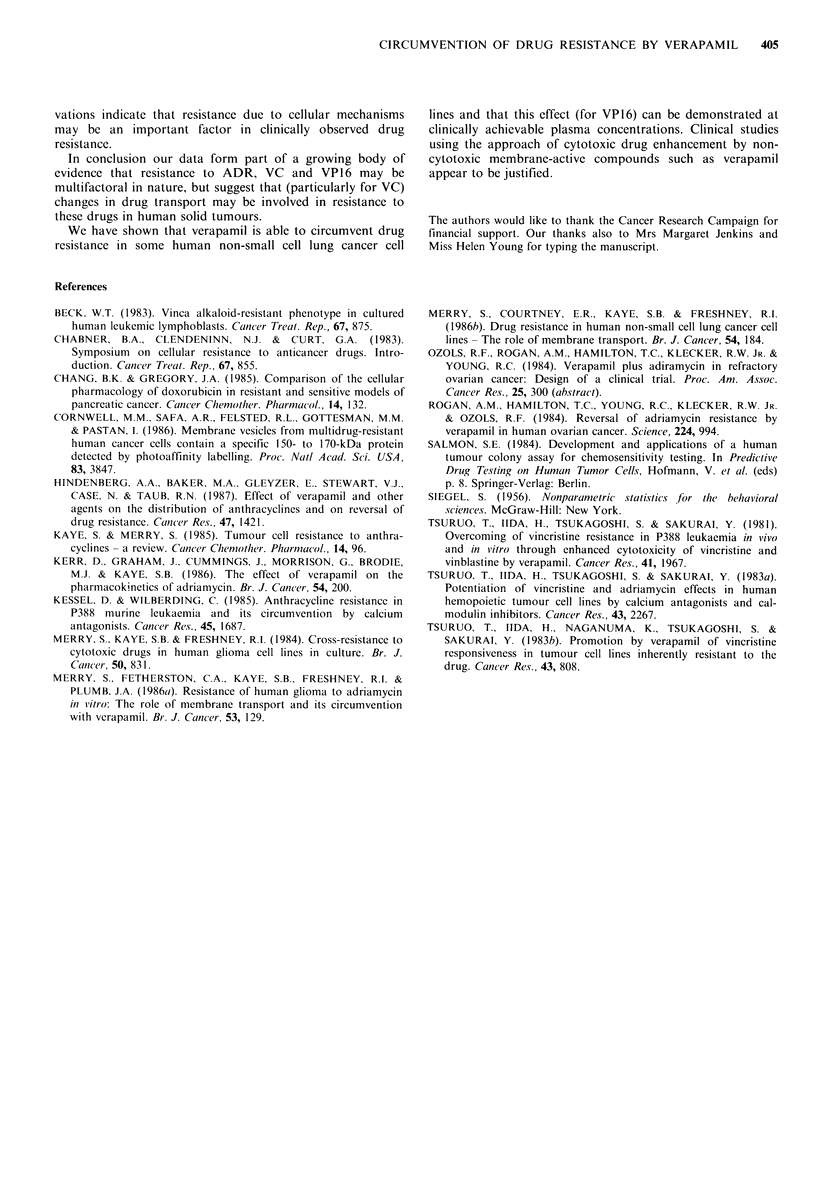# Circumvention of drug resistance in human non-small cell lung cancer in vitro by verapamil.

**DOI:** 10.1038/bjc.1987.214

**Published:** 1987-10

**Authors:** S. Merry, E. R. Courtney, C. A. Fetherston, S. B. Kaye, R. I. Freshney

**Affiliations:** CRC Department of Medical Oncology, University of Glasgow, Scotland, UK.

## Abstract

The sensitivity of 7 human non-small cell lung cancer cell lines to each of 7 cytotoxic drugs was determined. None of the cell lines used in these experiments had been previously exposed to cytotoxic drugs in vitro. A pattern of cross-resistance (P less than 0.05) between the drugs adriamycin (ADR), vincristine (VC) and etoposide (VP16) was noted similar to that seen in other models. The calcium antagonist verapamil (6.6 microM) was shown to increase sensitivity (up to 29-fold) to ADR, VC or VP16 in 5 cell lines. For 2 of the cell lines (A549 and WIL) 2.2 microM verapamil increased VP16 cytotoxicity (up to 4-fold). Drug accumulation studies in 2 cell lines (A549 and SK-MES-1) showed that 6.6 microM verapamil increased intracellular levels of VC up to 4-fold with the greatest increase seen in the cell line (SK-MES-1) for which verapamil produced the greatest increase in cytotoxicity (10-fold). For ADR and VP16 increases in drug accumulation were smaller (up to 1.6-fold). Our data support a potential clinical role for verapamil in overcoming cytotoxic drug resistance in human lung cancer.


*Br. J. Cancer* (1987), **56**, 401–405 © The Macmillan Press Ltd., 1987

### Circumvention of drug resistance in human non-small cell lung cancer *in vitro* by verapamil

S. Merry, E.R. Courtney, C.A. Fetherston, S.B. Kaye & R.I. Freshney

*CRC Department of Medical Oncology, University of Glasgow, 1 Horselethill Road, Glasgow G12 9LX, Scotland, UK.*

**Summary** The sensitivity of 7 human non-small cell lung cancer cell lines to each of 7 cytotoxic drugs was determined. None of the cell lines used in these experiments had been previously exposed to cytotoxic drugs *in vitro*. A pattern of cross-resistance ($P<0.05$) between the drugs adriamycin (ADR), vincristine (VC) and etoposide (VP16) was noted similar to that seen in other models. The calcium antagonist verapamil (6.6 $\mu$M) was shown to increase sensitivity (up to 29-fold) to ADR, VC or VP16 in 5 cell lines. For 2 of the cell lines (A549 and WIL) 2.2 $\mu$M verapamil increased VP16 cytotoxicity (up to 4-fold). Drug accumulation studies in 2 cell lines (A549 and SK-MES-1) showed that 6.6 $\mu$M verapamil increased intracellular levels of VC up to 4-fold with the greatest increase seen in the cell line (SK-MES-1) for which verapamil produced the greatest increase in cytotoxicity (10-fold). For ADR and VP16 increases in drug accumulation were smaller (up to 1.6-fold). Our data support a potential clinical role for verapamil in overcoming cytotoxic drug resistance in human lung cancer.

Many of the experimental models of drug resistance rely on the development of resistance *in vitro* through growth of cells in drug-containing medium or *in vivo* through treatment of mice bearing ascites or subcutaneous tumours. While these models have the advantage that they produce sensitive and resistant sublines of the same cell line the general relevance of these models to clinical drug resistance is yet to be established. In particular, using these models, cross-resistance between anthracyclines, vinca alkaloids, VP16 and antibiotics is often observed (see **Discussion**). Since certain therapeutic strategies (e.g. the use of calcium antagonists; Tsuruo *et al.*, 1981) have been shown to be effective in overcoming this resistance, the demonstration of a similar pattern of cross resistance in human tumour cell lines which have not been exposed to cytotoxic drugs in the laboratory would suggest that clinical trials employing similar strategies might be justified.

In this paper we use the term inherent resistance to describe resistance that has not been induced by exposure to cytotoxic drugs in experimental animals or *in vitro*.

For the drugs adriamycin (ADR) and vincristine (VC) Tsuruo *et al.* (1983*a,b*) have shown that the inherent resistance of both human haemopoietic and murine tumour lines can be circumvented by the calcium antagonist verapamil. Merry *et al.* (1986*a*) have also shown that inherent resistance to ADR in human glioma cell lines can be overcome by verapamil and that this is associated with increased intracellular drug levels. Similar results have also been obtained by Rogan *et al.* (1984) using human ovarian cancer cell lines.

In this paper we present data on the cross-sensitivity of a panel of human non-small cell lung cancer cell lines to some commonly-used cytotoxic drugs. We also report data on the use of verapamil as a means of circumventing resistance in human lung cancer which suggest that clinical studies employing this approach may be justified.

### Materials and methods

Seven established human non-small cell lung cancer cell lines were used in this study. Their sources, pathological details and (where available) the treatment undergone by the patient prior to the establishment of the cell line are shown in Table I. All cell lines grew as monolayer cultures.

The standard growth medium consisted of a mixture of equal volumes of Ham's F10 and Dulbecco's Modified Eagle's Medium supplemented with 10% foetal calf serum and with a gas phase of 2% $CO_2$. In some cases this medium was also supplemented with 50 $\mu g\,ml^{-1}$ gentamycin sulphate.

The drugs used in this study were adriamycin (ADR, Farmitalia Carlo Erba Ltd, Barnet, Herts, UK), actinomycin D (AD, trade name Lyovac Cosmegen, Merk Sharp and Dohme International, Rahway, NJ, USA), vincristine (VC, trade name Oncovin, Eli Lilly and Co Ltd, Basingstoke, UK), VP16 (etoposide, VP16-213, trade name Vepesid, Bristol Myers Pharmaceuticals, Slough, UK), L-penylalanine mustard (L-PAM, melphalan, trade name Alkeran, The Wellcome Foundation Ltd, London, UK), mitomycin C (MC, Kyowa Hakko Kogyo Co Ltd, Tokyo, Japan), 5-fluorouracil (5FU, Roche Products Ltd, Welwyn Garden City, Herts, UK) and verapamil (trade name Cordilox IV, Abbot Laboratories Ltd, Queenborough, Kent, UK). The drugs were solubilised according to the manufacturers' instructions for injection and then stored as frozen aliquots at −20°C until required (generally no longer than 2 months after freezing). In the case of L-PAM care was taken to ensure these operations were carried out within 30 min because of the instability of the drug in solution. L-4,5-[$^3$H]-leucine, [14-$^{14}$C]ADR and [G-$^3$H]VC were obtained from Amersham International Plc (Amersham, Bucks, UK). [G-$^3$H]-VP16 was prepared as a custom synthesis (Amersham International Plc) by tritium labelling a sample of VP16 powder (Bristol-Myers Pharmaceuticals). The final product was purified in our laboratories by thin layer chromatography. $^3$H-leucine and $^3$H-VC were stored according to the manufacturers' instructions. $^{14}$C-ADR and $^3$H-VP16 were stored in methanolic solution at −20°C.

### *Cytotoxicity assay*

The method used has been described previously (Merry *et al.*, 1984). Briefly, exponentially growing cultures in 96-well Linbro microtitration plates were treated with serial dilutions of drug (or drug combined with verapamil) for 72 h (with drug replacement at 24 and 48 h), followed by a recovery period in the absence of drug of 120 h (with medium replacement at 72 and 96 h). Viability of the cultures was then assessed as the incorporation of [$^3$H]-leucine into the trichloroacetic acid-insoluble fraction of the cells. The incorporation was expressed as a percentage of control cultures and the concentration of drug that inhibited protein synthesis by 50% ($ID_{50}$) determined. Cell counts (model $ZB_1$ Coulter counter) of trypsinised replicate cultures were used to determine population doubling time and to ensure that

Correspondence: S. Merry.
Received 1 December 1986; and in revised form, 12 May 1987.

**Table I** Non-small cell lung cancer cell lines

| *Cell line* | *Pathology* | *Source* | *Prior clinical treatment* |
|---|---|---|---|
| Calu-3 | Adenocarcinoma | American Type Culture Collection, USA | Yes[a] |
| NCI-H23 | Adenocarcinoma | National Cancer Institute, USA | None |
| NCI-H125 | Adenocarcinoma | National Cancer Institute, USA | None |
| WIL | Adenocarcinoma | Ludwig Institute, Sutton, UK | Not reported |
| L-DAN | Squamous carcinoma | Medical Oncology, Glasgow, UK | None |
| SK-MES-1 | Squamous carcinoma | American Type Culture Collection, USA | Not reported |
| A549 | Bronchiolo-alveolar carcinoma | American Type Culture Collection, USA | Not reported |

[a]Cytotoxan, bleomycin, adriamycin.

control cultures remained in exponential growth throughout the period of the experiment. In all experiments the period of drug exposure (72 h) exceeded one population doubling time.

*Drug uptake assay*

The method used has been described previously (Merry *et al.*, 1986*a*). Briefly, $7.9 \times 10^4$ exponentially growing cells ($10^5$ cells $cm^{-2}$) were seeded into 10 mm diameter soda glass specimen tubes. After 72 h the cell monolayer was washed and 0.2 ml of Hanks' balanced salts solution (adjusted to pH 7.4) supplemented with glucose (6.1 mM final concentration), MEM vitamins (Gibco Ltd, Paisley, UK), 4.5 mM $NaHCO_3$, 6.6 $\mu$M verapamil (in some cases) and 1 $\mu$Ci $ml^{-1}$ radiolabelled drug (ADR, VC or VP16). The final concentrations of ADR, VC and VP16 were 20 $\mu$M, 0.13 $\mu$M and 1.0 $\mu$M respectively. Replicate tubes (4 per time point) were then incubated at 37°C for 0, 30, 60 or 90 min before the cell monolayer was washed with phosphate-buffered saline (0°C) and a further 0.2 ml of drug-free Hanks' balanced salts solution (supplemented as above and without verapamil) was added. After a further incubation (30 min, 37°C) *unbound drug* was determined using liquid scintallation counting as the amount of radioactivity released into the supernatant medium. *Bound drug* was determined by counting the residual radioactivity of the washed, solubilised cell monolayer. In all experiments cell counts (model $ZB_1$ Coulter counter) from replicate tubes were used to express the results as pmol drug taken up $10^{-6}$ cells.

### Results

*Drug sensitivity experiments*

Table II shows the results of our drug sensitivity experiments. Where duplicate determinations were carried out the range of the results obtained are shown in the table. All replicate determinations of $ID_{50}$ fell within a 2.5-fold range.

Table III shows the cell lines ranked in order of sensitivity of the cytotoxic drugs. In the case of duplicate determinations the mean of the results was used to assign ranking position. Statistical analysis of these data was carried out using Kendall's coefficient of concordance (W) (Siegel, 1956). The null hypothesis is that there is no correlation between the ranking of sensitivity of the 7 cell lines. The value of W for the whole table is 0.46 ($P<0.01$). Although this value is highly significant, it can be increased to 0.60 by the exclusion of L-PAM, MC and 5FU from the calculations. These results indicate a pattern of cross-resistance between the drugs ADR, AD, VC and VP16.

**Table III** Drug sensitivity of NSCLC cell lines – ranked from most sensitive (1) to most resistant (7)

| *Cell line* | *ADR* | *AD* | *VC* | *VP16* | *L-PAM* | *MC* | *5FU* |
|---|---|---|---|---|---|---|---|
| NCI-H23 | 1 | 1 | 2 | 2 | 2 | 5 | 3 |
| Calu-3 | 2 | 3 | 1 | 1 | 3 | 2 | 2 |
| NCI-H125 | 3 | 2 | 6 | 7 | 1 | 1 | 1 |
| SK-MES-1 | 4 | 7 | 4 | 4 | 4 | 6 | 7 |
| L-DAN | 5 | 5 | 3 | 5 | 5 | 4 | 5 |
| WIL | 6 | 6 | 7 | 3 | 7 | 3 | 6 |
| A549 | 7 | 4 | 5 | 6 | 6 | 7 | 4 |

*Effects of verapamil on drug sensitivity*

Table IV shows the effect of 6.6 $\mu$M verapamil (added during the period of cytotoxic drug treatment only) on ADR, VC and VP16 sensitivity. Only 5 of the 7 cell lines were included in this study since 6.6 $\mu$M verapamil was found to be appreciably toxic to NCI-H23 and Calu-3 (Table II). Where duplicate experiments were carried out, the range of results is shown. In these cases the variability of the results was always less than 2.3-fold.

In 10 out of 15 cases (i.e. 3 drugs and 5 cell lines) Table IV shows an increase in cytotoxicity produced by this non-cytotoxic concentration of verapamil. This enhancement particularly in the case of ADR and VC is most pronounced in those cell lines which were most resistant to drug alone, i.e. WIL, A549 and L-DAN in the case of ADR; WIL, NCI-H125, A549 and SK-MES-1 in the case of VC.

**Table II** Cytotoxicity Data

| | *Drug $ID_{50}$* | | | | | | | |
|---|---|---|---|---|---|---|---|---|
| *Cell line* | *ADR* ($\times 10^{-9}$ M) | *AD* ($\times 10^{-10}$ M) | *VC* ($\times 10^{-9}$ M) | *VP16* ($\times 10^{-8}$ M) | *L-PAM* ($\times 10^{-7}$ M) | *MC* ($\times 10^{-11}$ M) | *5FU* ($\times 10^{-7}$ M) | *Verapamil* ($\times 10^{-6}$ M) |
| NCI-H23 | 0.002–0.005 | 9.3 | 1.9 | 33 | 0.8–2.0 | 79 | 8 | 4.5 |
| Calu-3 | 2.7 | 20 | 1.6 | 2.3 | 7.8 | 20 | 7.4 | 1.0 |
| NCI-H125 | 9.0 | 12 | 40–40 | 381 | 0.4 | 17 | 3.8–3.8 | 60 |
| SK-MES-1 | 48–53 | 60 | 13–22 | 81–120 | 24 | 100 | 86 | >55 |
| L-DAN | 34–70 | 50 | 17–17 | 92–110 | 84 | 60 | 42 | >55 |
| WIL | 168 | 55 | 136 | 70–83 | 100 | 30 | 69 | >55 |
| A549 | 204–228 | 20–40 | 27–28 | 360 | 87 | 200 | 15 | >55 |

**Table IV** Increase in drug sensitivity produced by 6.6 μM verapamil

| *Cell line* | *ADR* | *VC* | *VP16* |
|---|---|---|---|
| NCI-H125 | x 0.8[a] | x 25.9– x 32.0 | x 2.5 |
| SK-MES-1 | x 2.7– x 5.3 | x 10.0 | x 1.0 |
| L-DAN | x 2.4– x 5.0 | x 1.1 | x 1.1– x 1.3 |
| WIL | x 8.4 | x 2.6– x 4.0 | x 11.0– x 13.9 |
| A549 | x 2.1– x 4.8 | x 5.2– x 5.4 | x 5.3 |

[a]Fold change in $ID_{50}$.

The effect of 2.2 μM and 0.7 μM verapamil on ADR, VC and VP16 cytotoxicity for the cell lines WIL and A459 was also determined. These cell lines were chosen for this study since an increase in sensitivity to all 3 cytotoxic drugs was noted by 6.6 μM verapamil. Duplicate determinations were carried out in all cases and the variability of the duplicates was always less than 1.7-fold.

Verapamil at 2.2 μM increased VP16 cytotoxicity for both WIL (4.0-fold change in $ID_{50}$) and A549 (3.3-fold change in $ID_{50}$) i.e. 32% (WIL) and 62% (A549) of that produced by 6.6 μM verapamil. In no other case was an increase in sensitivity noted (data not shown).

*Drug uptake experiments*

In preliminary experiments with each of the drug combinations and using identical conditions to those of the drug uptake studies, cell viability (as determined by trypan blue exclusion) and cell loss from the monolayer (as determined by counting cell number in representative microscope fields) were reduced by <5% for both A549 and SK-MES-1 (data not shown).

In the drug uptake studies *unbound* drug was determined as that released during a 30 min incubation in glucose-containing medium. In control experiments (for each drug or drug combination) release of radioactivity under these conditions was shown to be initially rapid followed by a slower (apparently linear) release (data not shown). The initial phase of drug release reached completion at 30 min.

Figure 1 shows the effect of 6.6 μM verapamil on the uptake of VC by SK-MES-1. The general shape of the curves are typical of those obtained for all three drugs (ADR, VC and VP16) in both cell lines (A549 and SK-MES-1). In all cases uptake of *unbound* drug was close to plateau levels at 30 min (both in the absence and presence of verapamil), while levels of *bound* drug in the presence of verapamil were apparently still rising at 90 min. The data of the drug uptake experiments are shown in Table V. For ADR, VC and VP16 respectively A549 is 4.3, 1.6 and 3.6-fold more resistant than SK-MES-1 (data taken from Table II). The ratios (A549:SK-MES-1) of total drug levels at 90 min are however 0.8, 1.3 and 1.7 in the absence of verapamil for ADR, VC and VP16 respectively.

**Figure 1** Vincristine uptake by the cell line SK-MES-1. (a) Unbound drug; (b) bound drug. In the presence (○) and absence (●) of 6.6 μM verapamil. All experiments were carried out at 37°C using Hanks' balanced salt solution supplemented with glucose (6.1 mM final concentration), MEM vitamines and 0.13 μM $^3$H-vincristine. Error bars indicate mean ±s.e. Error bars are omitted when their range would be smaller than the size of the symbol used to indicate mean value.

Table VI summarises the data obtained in the drug uptake experiments and enables a comparison of this data with the effect of verapamil on cytotoxicity. For VC verapamil produced major increases in both cytotoxicity and drug accumulation. Furthermore for SK-MES-1 the increases produced by verapamil on cytotoxicity, drug accumulation and drug

**Table V** Effect of 6.6 μM verapamil on drug accumulation

| *Cell line* | | | *Drug accumulation at 90 min (pmol 10⁻⁶ cells)* ADR | VC | VP16 |
|---|---|---|---|---|---|
| A549 | – Verapamil | Unbound | 36.3±0.8 [a] | 9.0±0.5 | 12.4±0.3 |
| | | Bound | 61.6±2.3 | 11.0±0.1 | 9.8±0.1 |
| | + Verapamil | Unbound | 38.0±0.7 | 20.0±0.6 | 21.2±0.8 |
| | | Bound | 78.7±2.7 | 28.3±0.9 | 14.8±0.2 |
| SK-MES-1 | – Verapamil | Unbound | 41.2±3.2 | 7.2±0.5 | 6.8±0.3 |
| | | Bound | 85.5±10.1 | 8.4±0.3 | 7.3±0.1 |
| | + Verapamil | Unbound | 47.3±2.8 | 23.5±0.5 | 8.8±0.3 |
| | | Bound | 117.0±13.1 | 44.6±1.2 | 9.8±0.2 |

[a]Mean ±s.e. (n=4).

Table VI Comparison of the effects of 6.6 μM verapamil on drug sensitivity and drug accumulation

| Cell line | | ADR | VC | VP16 |
|---|---|---|---|---|
| A549 | Sensitivity | ×3.5[a] | ×5.3 | ×5.3 |
| | Transport | ×1.2[b] | ×2.4 | ×1.6 |
| | Binding | ×1.2[c] | ×1.2 | ×0.9 |
| SK-MES-1 | Sensitivity | ×4.0 | ×10.0 | ×1.0 |
| | Transport | ×1.3 | ×4.4 | ×1.3 |
| | Binding | ×1.2 | ×1.6 | ×1.0 |

[a]Fold change in $ID_{50}$ (where duplicate determinations were made, the figure given in the mean value); [b]Fold change in total drug accumulation (bound plus unbound) at 90 min; [c]Fold change in ratio bound:unbound drug at 90 min.

binding were all ~2-fold those seen in A549. For ADR and VP16 the direct relationship between drug accumulation and sensitivity was not as clear since the increases in drug accumulation and binding were relatively small. For ADR, verapamil did however, produce increases in drug uptake, drug binding, and drug sensitivity in both cell lines. For VP16 in the case of cell line A549 the increase in cytotoxicity produced by verapamil is associated with increased drug uptake and drug binding. Increased drug uptake was also seen in the cell line SK-MES-1 where no effect of verapamil on cytotoxicity was noted. In both A549 and SK-MES-1 verapamil did not increase the ratio bound:unbound VP16.

### Discussion

We have examined the sensitivity of a panel of 7 non-small cell lung cancer cell lines to 7 cytotoxic drugs. None of the cell lines (4 adenocarcinomas, 2 squamous carcinomas and 1 bronchiolo-alveolar carcinoma) had been exposed to cytotoxic drugs *in vitro*. A pattern of cross-resistance (termed pleiotropic drug resistance, PDR) to ADR, VC and VP16 was found similar to that described in animal models (for review see Kaye & Merry, 1985). PDR has also been described in human haemopoietic tumour cell lines (Beck, 1983), human glioma cell lines (Merry *et al.*, 1984) and human small cell lung cancer cell lines (Shoemaker *et al.*, 1983). The cross-resistance data described here thus provide further evidence that PDR may be a general phenomenon in cell lines derived from human tumours, although the clinical relevance of PDR still remains to be established.

PDR has two important characteristics; (a) altered membrane transport (possibly enhanced drug efflux) has been postulated as the major factor underlying this resistance and (b) in some experimental models PDR has been circumvented by calcium antagonists such as verapamil (for review see Chabner *et al.*, 1983).

We have studied the effect of 6.6 μM verapamil on the cytotoxicity of ADR, VC and VP16 in 5 cell lines. This dose of verapamil has been previously used to potentiate the effects of ADR and VC in human haemopoietic tumour cell lines (Tsuruo *et al.*, 1983*a*) and of ADR in human ovarian tumour cell lines (Rogan *et al.*, 1984). This non-cytotoxic concentration of verapamil was able to potentiate the effect of the 3 cytotoxic drugs in some of the cell lines (up to 29-fold) with, particularly for ADR and VP16, potentiation in the most resistant cell lines.

Plasma levels of up to 10 μM verapamil may be achieved clinically by i.v. infusion (Ozols *et al.*, 1984), but these are associated with significant cardiovascular toxicity. Levels of 1–3 μM verapamil may be achieved in cancer patients using a daily oral dose of 480 mg per day (Kerr *et al.*, 1986), with minimal toxicity. In order to assess the efficacy of these lower dose levels we investigated the effect of 2.2 μM and 0.7 μM verapamil on ADR, VC and VP16 cytotoxicity using 2 cell lines (A549 and WIL) in which 6.6 μM verapamil potentiated the effect of all 3 drugs.

Verpamil did not enhance sensitivity to ADR and VC at these concentrations, but we did observe increased VP16 cytotoxicity at 2.2 μM verapamil in both cell lines. We have confirmed this observation *in vivo* in separate experiments which indicate that verapamil (at murine plasma levels of 1.6 μM) is able to increase the sensitivity of WIL xenografts to VP16 (Merry *et al.*, 1986*b*).

In the clinical trial of Kerr *et al.* (1986) patients with small cell lung cancer were treated with 4-day chemotherapy and 5 days of oral verapamil (480 mg $day^{-1}$) and plasma levels of 1.5 μM verapamil were obtained. In addition, levels of the major metabolite norverapamil were measured at 1.5 μM. Our preliminary laboratory studies investigating the biological activity of norverapamil in circumventing drug resistance indicate that it has approximately equal activity to verapamil (unpublished data).

Using two cell lines (A549 and SK-MES-1) we have also investigated the effects of 6.6 μM verapamil on ADR, VC and VP16 accumulation and binding. It was a common finding for both cell lines with ADR and VC that verapamil increased the ratio of bound:unbound drug. These data are consistent with the observations of Hindenberg *et al.* (1987) that, for ADR, verapamil displaces drug from the hydrophobic into the hydrophilic compartment of the cell. Drug within the hydrophilic compartment is potentially less readily released than lipid associated drug. Furthermore a recent study by Cornwell *et al.* (1986) has shown that verapamil is able to directly modulate the vinblastine photo-affinity labelling of a 170 kDa glycoprotein in membrane vesicles from multidrug resistant cells.

For VC a clear relationship emerged between the effects of verapamil on sensitivity, drug accumulation and binding (Table VI). This suggests that (at least in these two cell lines) the major mechanism by which verapamil increases cytotoxicity to VC may be by influencing drug transport and/or binding. For ADR and VP16 smaller increases in drug accumulation were seen. These increases are associated with increased sensitivity to ADR (in both cell lines) and VP16 (in A549). In SK-MES-1, however, verapamil increased VP16 accumulation, but had no effect on cytotoxicity. Drug accumulation may not be rate-limiting for cytotoxicity in this particular case due to saturation of the intracellular binding sites at which VP16 acts.

In previous study (Merry *et al.*, 1986*a*) using human glioma cell lines verapamil (13 μM) produced increases in ADR uptake, binding and sensitivity in resistant cell lines. The increases were of equivalent size to those seen in this study.

Verapamil might also be enhancing drug sensitivity by a mechanism or mechanisms unrelated to total intracellular drug accumulation and binding. The presence of additional mechanisms of resistance is indicated by the observation that for VC and VP16 the drug sensitivity data (Table II) show A549 to be respectively 1.6 and 3.6-fold more resistant than SK-MES-1 while in the drug uptake experiments (Table VI) accumulation of drug was greatest in A549.

While in this study reduced ADR accumulation was noted in A549 (resistant) compared to SK-MES-1 (sensitive) a lack of correlation between sensitivity and uptake has been reported for rodent pancreatic carcinoma cell lines (Chang & Gregory, 1985) and human glioma cell lines (Merry *et al.*, 1986*a*). Kessel and Wilbarding (1985) have also reported that differences in ADR sensitivity between 2 sublines of P388 muring leukaemia could not be totally accounted for by differences in drug accumulation.

It is also recognised that clinical resistance to cytotoxic drugs may occur in tumours by mechanisms not involving biochemical changes within individual tumour cells. Examples of such mechanisms might be a reduction in tumour blood supply (leading to decreased entry of cytotoxic drug into the tumour) or increased cytotoxic drug degradation at a site other than the tumour (e.g. the liver). Such factors may also occur in combination with cellular factors. Nevertheless studies in several human solid tumour types have shown that the occurrence of drug resistance in the clinic is associated with the presence of drug-resistant clonogenic cells in biopsy specimens (Salmon, 1984). These observations indicate that resistance due to cellular mechanisms may be an important factor in clinically observed drug resistance.

In conclusion our data form part of a growing body of evidence that resistance to ADR, VC and VP16 may be multifactoral in nature, but suggest that (particularly for VC) changes in drug transport may be involved in resistance to these drugs in human solid tumours.

We have shown that verapamil is able to circumvent drug resistance in some human non-small cell lung cancer cell lines and that this effect (for VP16) can be demonstrated at clinically achievable plasma concentrations. Clinical studies using the approach of cytotoxic drug enhancement by non-cytotoxic membrane-active compounds such as verapamil appear to be justified.

The authors would like to thank the Cancer Research Campaign for financial support. Our thanks also to Mrs Margaret Jenkins and Miss Helen Young for typing the manuscript.

### References

BECK, W.T. (1983). Vinca alkaloid-resistant phenotype in cultured human leukemic lymphoblasts. *Cancer Treat. Rep.*, **67**, 875.

CHABNER, B.A., CLENDENINN, N.J. & CURT, G.A. (1983). Symposium on cellular resistance to anticancer drugs. Introduction. *Cancer Treat. Rep.*, **67**, 855.

CHANG, B.K. & GREGORY, J.A. (1985). Comparison of the cellular pharmacology of doxorubicin in resistant and sensitive models of pancreatic cancer. *Cancer Chemother. Pharmacol.*, **14**, 132.

CORNWELL, M.M., SAFA, A.R., FELSTED, R.L., GOTTESMAN, M.M. & PASTAN, I. (1986). Membrane vesicles from multidrug-resistant human cancer cells contain a specific 150- to 170-kDa protein detected by photoaffinity labelling. *Proc. Natl Acad. Sci. USA*, **83**, 3847.

HINDENBERG, A.A., BAKER, M.A., GLEYZER, E., STEWART, V.J., CASE, N. & TAUB, R.N. (1987). Effect of verapamil and other agents on the distribution of anthracyclines and on reversal of drug resistance. *Cancer Res.*, **47**, 1421.

KAYE, S. & MERRY, S. (1985). Tumour cell resistance to anthracyclines – a review. *Cancer Chemother. Pharmacol.*, **14**, 96.

KERR, D., GRAHAM, J., CUMMINGS, J., MORRISON, G., BRODIE, M.J. & KAYE, S.B. (1986). The effect of verapamil on the pharmacokinetics of adriamycin. *Br. J. Cancer*, **54**, 200.

KESSEL, D. & WILBERDING, C. (1985). Anthracycline resistance in P388 murine leukaemia and its circumvention by calcium antagonists. *Cancer Res.*, **45**, 1687.

MERRY, S., KAYE, S.B. & FRESHNEY, R.I. (1984). Cross-resistance to cytotoxic drugs in human glioma cell lines in culture. *Br. J. Cancer*, **50**, 831.

MERRY, S., FETHERSTON, C.A., KAYE, S.B., FRESHNEY, R.I. & PLUMB, J.A. (1986*a*). Resistance of human glioma to adriamycin *in vitro*: The role of membrane transport and its circumvention with verapamil. *Br. J. Cancer*, **53**, 129.

MERRY, S., COURTNEY, E.R., KAYE, S.B. & FRESHNEY, R.I. (1986*b*). Drug resistance in human non-small cell lung cancer cell lines – The role of membrane transport. *Br. J. Cancer*, **54**, 184.

OZOLS, R.F., ROGAN, A.M., HAMILTON, T.C., KLECKER, R.W. JR. & YOUNG, R.C. (1984). Verapamil plus adiramycin in refractory ovarian cancer: Design of a clinical trial. *Proc. Am. Assoc. Cancer Res.*, **25**, 300 (*abstract*).

ROGAN, A.M., HAMILTON, T.C., YOUNG, R.C., KLECKER, R.W. JR. & OZOLS, R.F. (1984). Reversal of adriamycin resistance by verapamil in human ovarian cancer. *Science*, **224**, 994.

SALMON, S.E. (1984). Development and applications of a human tumour colony assay for chemosensitivity testing. In *Predictive Drug Testing on Human Tumor Cells*, Hofmann, V. *et al.* (eds) p. 8. Springer-Verlag: Berlin.

SIEGEL, S. (1956). *Nonparametric statistics for the behavioral sciences*. McGraw-Hill: New York.

TSURUO, T., IIDA, H., TSUKAGOSHI, S. & SAKURAI, Y. (1981). Overcoming of vincristine resistance in P388 leukaemia *in vivo* and *in vitro* through enhanced cytotoxicity of vincristine and vinblastine by verapamil. *Cancer Res.*, **41**, 1967.

TSURUO, T., IIDA, H., TSUKAGOSHI, S. & SAKURAI, Y. (1983*a*). Potentiation of vincristine and adriamycin effects in human hemopoietic tumour cell lines by calcium antagonists and calmodulin inhibitors. *Cancer Res.*, **43**, 2267.

TSURUO, T., IIDA, H., NAGANUMA, K., TSUKAGOSHI, S. & SAKURAI, Y. (1983*b*). Promotion by verapamil of vincristine responsiveness in tumour cell lines inherently resistant to the drug. *Cancer Res.*, **43**, 808.